# T1-weighted MRI-driven Brain Age Estimation in Alzheimer’s Disease and Parkinson’s Disease

**DOI:** 10.14336/AD.2019.0617

**Published:** 2019-06-17

**Authors:** Iman Beheshti, Shiwangi Mishra, Daichi Sone, Pritee Khanna, Hiroshi Matsuda

**Affiliations:** ^1^Integrative Brain Imaging Center, National Center of Neurology and Psychiatry, Tokyo, Japan.; ^2^PDPM Indian Institute of Information Technology, Design and Manufacturing, Jabalpur, India.

**Keywords:** brain age, T1-weighted MRI, estimation, Alzheimer's disease, Parkinson’s disease

## Abstract

Neuroimaging-driven brain age estimation has introduced a robust (reliable and heritable) biomarker for detecting and monitoring neurodegenerative diseases. Here, we computed and compared brain age in Alzheimer’s disease (AD) and Parkinson’s disease (PD) patients using an advanced machine learning procedure involving T1-weighted MRI scans and gray matter (GM) and white matter (WM) models. Brain age estimation frameworks were built using 839 healthy individuals and then the brain estimated age difference (Brain-EAD: chronological age subtracted from brain estimated age) was assessed in a large sample of PD patients (n = 160) and AD patients (n = 129), respectively. The mean Brain-EADs for GM were +9.29 ± 6.43 years for AD patients versus +1.50 ± 6.03 years for PD patients. For WM, the mean Brain-EADs were +8.85 ± 6.62 years for AD patients versus +2.47 ± 5.85 years for PD patients. In addition, PD patients showed a significantly higher WM Brain-EAD than GM Brain-EAD. In a direct comparison between PD and AD patients, we observed significantly higher Brain-EAD values in AD patients for both GM and WM. A comparison of the Brain-EAD between PD and AD patients revealed that AD patients may have a significantly “older-appearing” brain than PD patients.

Neurodegenerative diseases are progressive disorders that primarily affect the neurons in the human brain [1]. Many neurodegenerative diseases such as Alzheimer’s disease (AD) and Parkinson’s disease (PD) are associated by neuronal loss and structural changes in different areas of the brain [2]. These diseases are marked by cognitive impairment resulting in memory loss, language impairment, and reduced mental functioning in AD and loss of motor function, movement disorder, tremors, and stiffness in PD [3]. The cognitive impairment occurring in these diseases could be progressive and results in behavioral and anatomical changes [4]. The normal aging process gradually leads to similar deficits, including cognitive decline, vision loss, and balance issues, which are analogous to the neurodegenerative disorder-related structural variations but delayed by a number of years [5]. Normal aging can also be considered progression along a temporal trajectory that ultimately culminates in an individual’s death. However, because chronic diseases such as AD and PD affect the brain neurons and result in brain atrophy, this trajectory of normal aging is altered, modifying the brain aging process [6].

AD and PD show significant clinical and pathological overlap, with similar and yet undetermined etiological changes possibly found in both AD and PD [7]. AD is associated by neuronal loss, extracellular senile plaques containing the peptide β amyloid, and neurofibrillary tangles composed of a protein called tau [8]. PD is linked by some pathological features such as loss of neurons in the substantia nigra and elsewhere and the presence of ubiquitinated protein deposits in the cytoplasm of neurons (Lewy bodies) [9, 10]. Research has revealed an association between AD and PD and their overlapping clinical and pathological profiles suggest similarities in their pathogeneses. Loss of neurons in the nucleus basalis of Meynert and the locus coeruleus, the principal cholinergic and noradrenergic nuclei projecting to the cortex, respectively, have been observed in both PD and AD. Neurons are lost in the midbrain even in AD patients with no obvious Lewy bodies (or in patients that have AD) [11]. In addition, neurofibrillary tangles are present in dopaminergic neurons of the substantia nigra in AD. Neurological studies have also shown a decreased number of neurons in the zona compacta of the substantia nigra of AD patients. However, the pathology of PD and AD may differ in terms of the oculomotor neurons of the rostral midbrain where PD produces neuronal loss and Lewy body formation, which is not seen in AD [12].

Neuroimaging methods have been proposed to estimate an individual’s brain age based on the structural alterations and variations in brain regions [13]. The discrepancy between this age and the chronological age defines a highly reliable and heritable biomarker known as the brain estimated age difference (Brain-EAD: chronological age subtracted from brain estimated age). This method compares the age estimated by the system with the chronological age of the individual to determine the status of the brain. Estimates of brain age, derived using machine learning, have previously been used in a number of contexts [14-18]. Initially, this brain age model was applied to species-specific adaptations for experimental animal studies, including baboons [19] and rodents [20]. It has also been used to study brain maturation in childhood and adolescence and to compare preterm-born adolescents (born before the end of the 27th week of gestation) with adolescents born after the end of the 29^th^ week of gestation [21]; the preterm-born adolescents had delayed structural brain maturation. Brain age analysis of individuals with psychiatric disorders involving schizophrenia and bipolar 1 disorder revealed that brain age scores are increased by 2.6 years for schizophrenia but are unaltered for bipolar disorder [22]. In addition, nondemented individuals with type 2 diabetes mellitus were found to have a 4.6 year higher brain age than healthy individuals [23]. Lifestyle risk factors (e.g., hypertension, smoking, and alcohol intake), tumor necrosis factor levels, and common clinical outcomes, such as cognition or depression, are associated with higher brain age scores [24]. The brain age concept has recently been in investigating neurodegenerative disorders to enable the early diagnosis of AD and predict conversion from mild cognitive impairment (MCI) to AD [14]. For instance, the [14] employed an automatic brain age estimator for estimating the age of healthy individuals and a clinical sample from the ADNI dataset. They reported an MAE of 4.98 years for the estimated age of HC individuals and a mean Brain-EAD of +10 years for AD samples based on GM. The researchers in [25], conducted a brain-age framework to assess the accuracy of conversion prediction from MCI to AD through the mean of brain age score. According to this research, an accuracy value of up to 81% was achieved in estimating conversion from MCI to AD in MCI subjects using anatomical MRI data. These studies suggest a link between disease progression and aging. Furthermore, such studies have motivated the analysis of other neurodegenerative diseases.

With respect to clinical, pathological, and genetic similarities and differences between AD and PD patients [26, 27], we conducted this study to compute and compare the Brain-EAD values among AD and PD patients using a robust brain age estimation framework involving T1-weighted (T1w) magnetic resonance imaging (MRI) scans and multivariate machine learning, and the following hypotheses were assessed:

- PD patients have a higher WM Brain-EAD than GM Brain-EAD.
- There are significant Brain-EAD differences between PD and AD patients.
- AD patients have a significantly “older-appearing” brain compared with PD patients.

## MATERIALS AND METHODS

### Participants and MRI acquisition

A total of 1,128 T1w MRI scans were used from the IXI (http://brain-development.org/ixi-dataset/), Open Access Series of Imaging Studies (OASIS) (https://www.oasis-brains.org/), Alzheimer's Disease Neuroimaging Initiative (ADNI) (www.loni.ucla.edu/ADNI/), and Parkinson’s Progression Markers Initiative (PPMI) (www.ppmi-info.org) databases. The training dataset comprised 839 T1w MRI scans from healthy controls (HCs) (aged 35-90 years) obtained from the IXI, OASIS, ADNI, and PPMI datasets. The test set included 160 PD and 129 AD patients acquired from the PPMI and ADNI datasets, respectively.

Regarding the AD patients, the Mini-Mental State Examination (MMSE) and Geriatric Depression Scale (GDS) were considered clinical parameters in the statistical analysis. The clinical parameters of PD patients comprised disease duration, the Montreal Cognitive Assessment (MoCA) test score, GDS, Movement Disorder Society-Sponsored Revision of the Unified Parkinson's Disease Rating (MDS-UPDRS Total) Scale, MDS-UPDRS Part I, MDS-UPDRS Part I Patient Questionnaire, MDS-UPDRS Part II Patient Questionnaire, MDS-UPDRS Part III, Schwab & England (S&E) scale, cUPSIT), Scales for Outcomes in Parkinson's Disease-Autonomic Questionnaire (SCOPA-AUT), and specific binding ratios (SBRs) of the left/right caudate and left/right putamen. The study participants’ details are shown in Table 1.

**Table 1:** Characteristics of subjects in this study.

Training dataset					Test dataset	

Dataset	IXI	OASIS	ADNI	PPMI	ADNI	PPMI
Category	HC	HC	HC	HC	AD	PD
No of Subjects	408	103	227	101	129	160
Female/Male	238/170	78/25	110/117	37/64	64/65	64/96
Age (years)	56.48±12.07	67.81±12.85	75.96±5.04	60.24±10.02	71.64±5.81	64.53±6.98
MMSE	n/a	n/a	n/a	n/a	23.25±2.26	n/a
CDR	n/a	n/a	n/a	n/a	0.75±0.31	n/a
GDS	n/a	n/a	n/a	n/a	n/a	2.24±2.33
MoCA	n/a	n/a	n/a	n/a	n/a	26.91±2.40
MDS-UPDRS Total	n/a	n/a	n/a	n/a	n/a	31.97±13.13
MDS-UPDRS Part I	n/a	n/a	n/a	n/a	n/a	1.35±1.57
MDS-UPDRS Part I Patient questionnaire	n/a	n/a	n/a	n/a	n/a	4.05±2.79
MDS-UPDRS Part II Patient questionnaire	n/a	n/a	n/a	n/a	n/a	5.71±3.96
MDS-UPDRS Part III	n/a	n/a	n/a	n/a	n/a	20.85±8.72
S&E	n/a	n/a	n/a	n/a	n/a	93.78±5.65
UPSIT	n/a	n/a	n/a	n/a	n/a	21.01±8.19
SCOPA-AUT	n/a	n/a	n/a	n/a	n/a	9.61±5.68
SBR Left Caudate	n/a	n/a	n/a	n/a	n/a	1.88±0.60
SBR Right Caudate	n/a	n/a	n/a	n/a	n/a	1.87±0.58
SBR Left Putamen	n/a	n/a	n/a	n/a	n/a	0.77±0.31
SBR Right Putamen	n/a	n/a	n/a	n/a	n/a	0.79±0.31

Note: All data are presented in mean ± standard deviation mode. n/a =not available.

### Neuroimaging processing

Pre-processing of the T1w MRI scans was performed by using SPM (Statistical Parameter Mapping) v12 (http://www.fil.ion.ucl.ac.uk/spm) software. As described in [28], all of the T1w MRI scans were bias corrected and segmented into WM, GM, and cerebrospinal fluid components using a generative model. The WM and GM images of the training dataset (i.e., healthy individuals, n = 839) were used to create a DARTEL template [29], using SPM DARTEL. This non-linear deformation template formed by using high-dimensional normalization is used to create DARTEL warped images. All of the DARTEL warped images were registered with standard MNI (Montreal Neurological Imaging) space maps using linear affine transformation. Then, GM and WM images were smoothed with 4-mm smoothing kernels [28] followed by a 8-mm isotropic spatial resolution. This procedure generated, for each individual, 3,747 aligned and smoothed GM or WM voxel intensities that were used as MRI features.

### Brain age estimation and model validation

**Figure 1.**
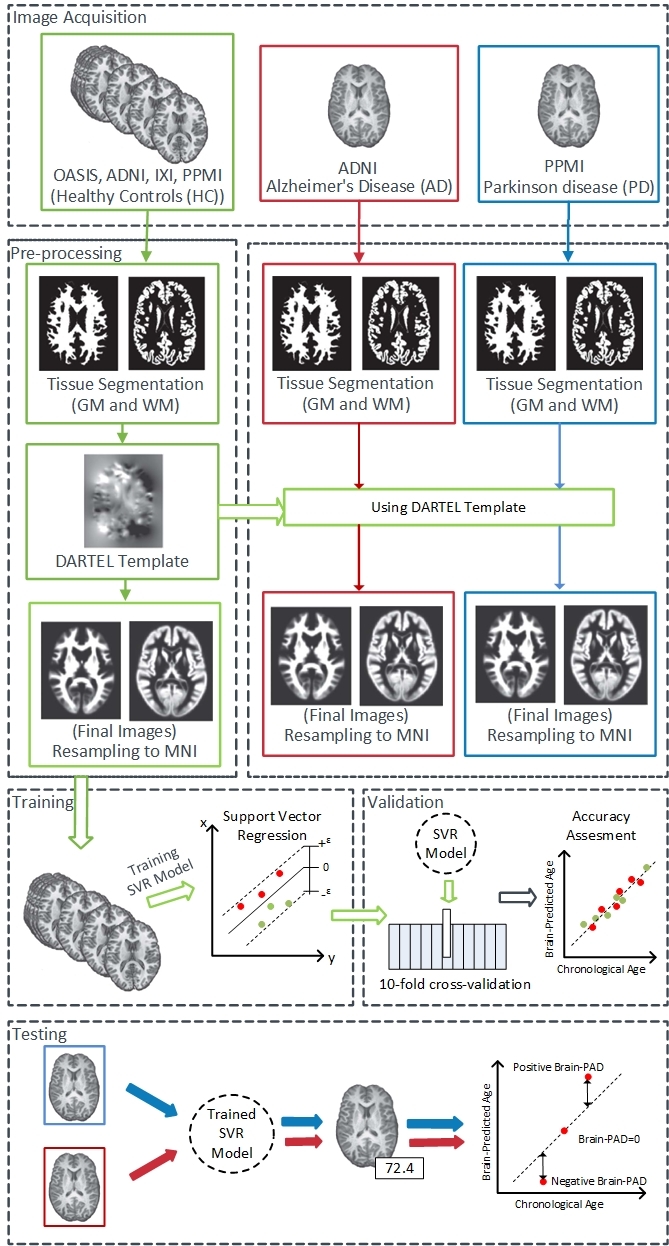
The pipeline of the T1w MRI-driven brain age estimation framework used in this study.

To build a brain age estimation framework, we used the support vector regression (SVR) algorithm [30] implemented in LIBSVM (www.csie.ntu.edu.tw/cjlin/libsvm/) library with a linear kernel and default setting, because it was shown to be a robust estimation model in a series of neuroimaging studies [31-35]. In each regression model, the GM and WM voxel intensities were considered independent variables with chronological age as the dependent variable. To assess the reliability of the brain age estimation framework, we used a 10-fold cross-validation on the basis of the GM and WM training set (i.e., healthy individuals, n = 839), separately. That is, the GM and WM training set was randomly split into 10 equal parts, with each, in turn, serving as the test set for the model fitted on the remaining 9/10th of the data. The accuracy of the brain age estimation frameworks was validated by use of the chronological age and the estimated brain age on the basis of the mean absolute error (MAE), root mean square error (RMSE), and correlation coefficient (between chronological and estimated brain age) through 10-fold cross-validation. The final brain age estimation framework was created using the entire training set (i.e., healthy individuals, n = 839) and then applied to AD patients (n = 129) and PD patients (n = 160) to estimate the brain ages through the GM and WM models. A high-level overview of the brain estimated age pipeline is provided in Figure 1.

**Figure 2.**
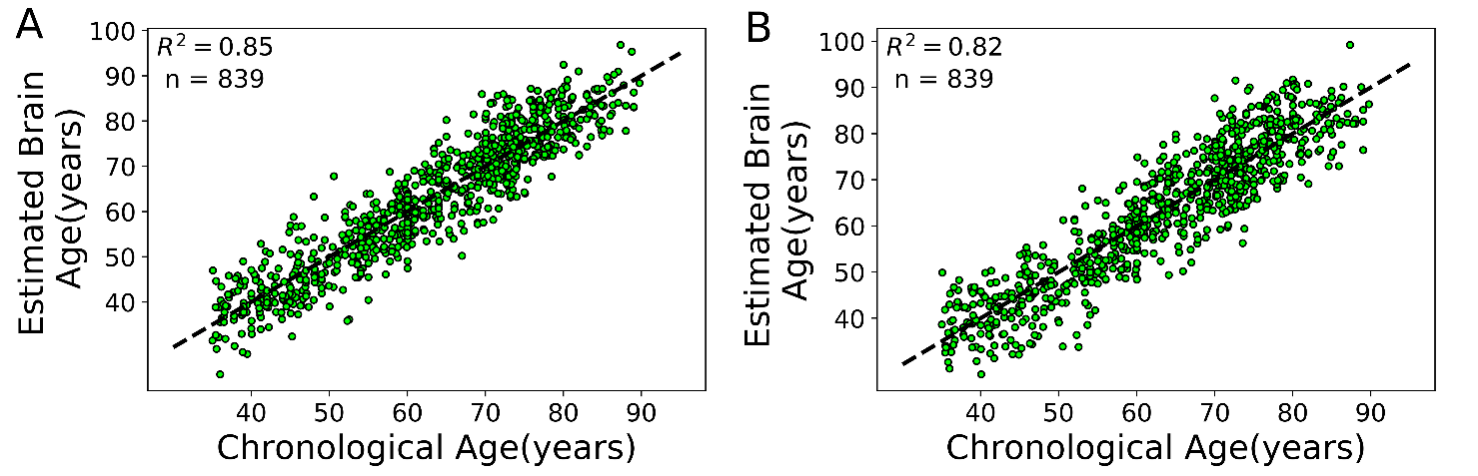
Chronological age versus estimated brain age in the training set (n = 839) through 10-fold cross-validation strategy. (A) GM model. (B) WM model. The identity line is illustrated with a dashed black line (y = x).

### Statistical analysis

To assess the mean Brain-EAD between groups, we used analysis of covariance (ANCOVA) with age and sex as covariates. Brain-EAD values were compared between GM and WM models in AD and PD patients using a paired-samples *t*-test. The association between the Brain-EAD and clinical parameters was analyzed using partial correlation with age and sex as covariates. Statistical analyses were conducted using SPSS (Statistical Package for Social Sciences) software version 16.0 (IBM, Armonk, NY) with *p* ˂ 0.05 considered significant.

**Table 2:** Performance of the proposed brain age framework in the training set.

	GM	WM
MAE (years)	4.38	4.85
RMSE (years)	5.46	6.06
Correlation (r)	0.92	0.91
Mean Brain-EAD [SD]	0.01 [5.46]	-0.05 [6.06]

## RESULTS

### Brain age estimation model in the training set

To assess the brain age in AD and PD patients, we built two independent brain age estimation frameworks in the training set (n = 839) using GM and WM models. For each model, we validated the proposed brain age estimation framework based on 10-fold cross-validation. The Brain-EAD values (mean ± SD) was -0.01 ± 5.46 years for GM and -0.05 ± 6.06 years for WM models in the training set. The details of the validation of the brain age framework are shown in Table 2 while the chronological ages versus predicted ages in the training set on the basis of the GM and WM models are shown in Figure 2. There was no significant correlation between Brain-EAD and chronological age for both GM and WM models (GM, r = 0.02, *p* = 0.51; WM, r= 0.04, *p* = 0.20) in the training set.

**Figure 3.**
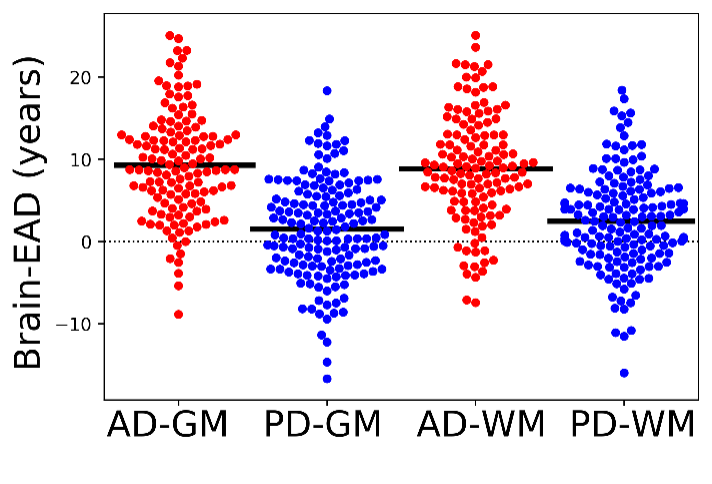
Comparison of Brain-EAD values between PD patients (blue spot) and AD patients (red spot) for the GM and WM models. The mean brain-EAD values of each group is illustrated with a solid black line. The reference line is illustrated with a dashed black line (y = 0).

### Brain-EAD in the AD and PD patients 

**Figure 4.**
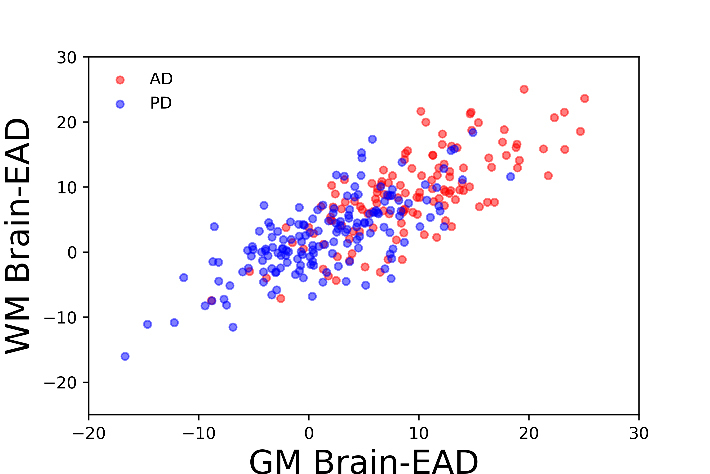
GM and WM Brain-EAD distributions for PD, and AD individuals.

To compute the Brain-EAD in AD and PD patients, we applied the GM and WM voxel intensities obtained from AD and PD patients to brain age estimation frameworks conducted by the training set. Grouped data plots showing the Brain-EAD (in years) for AD and PD patients are presented in Figure 3. The Brain-EAD values were as follows: PD patients (GM, +1.50 ± 6.03 years; WM, +2.47 ± 5.85 years), and AD patients (GM, +9.29 ± 6.43 years; WM, +8.85 ± 6.62 years). Both PD and AD groups showed significantly higher Brain-EAD values versus training set (i.e., brain-EAD of 0) for both the GM model (PD vs. training set: F = 3.32, *p* < 0.05; AD vs. training set: F = 102.47, *p* < 0.001; ANCOVA) and the WM model (PD vs. training set: F = 9.48, *p* < 0.001; AD vs. training set: F = 79.64, *p* < 0.001; ANCOVA). The distribution of GM and WM Brain-EAD values among the AD and PD of subjects is presented in Figure 4.

### Brain-EAD in PD versus AD

**Figure 5.**
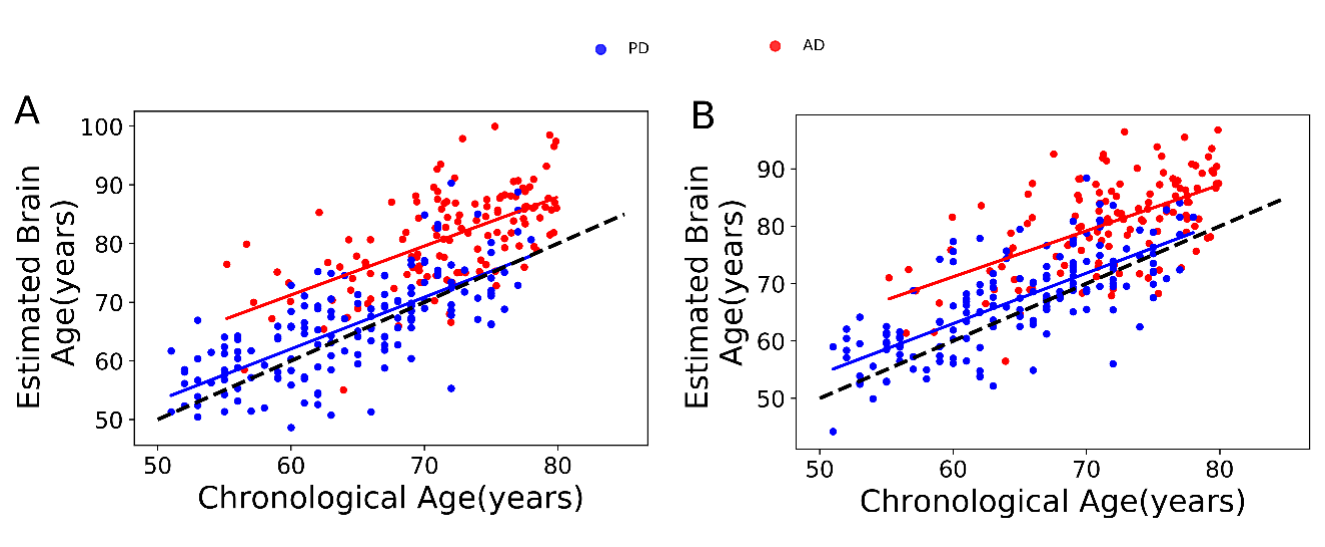
Chronological age versus estimated brain age among PD patients (blue spot, blue regression line) and AD patients (red spot, red regression line). (A) GM model. (B) WM model. The identity line is illustrated with a dashed black line (y = x).

**Figure 6.**
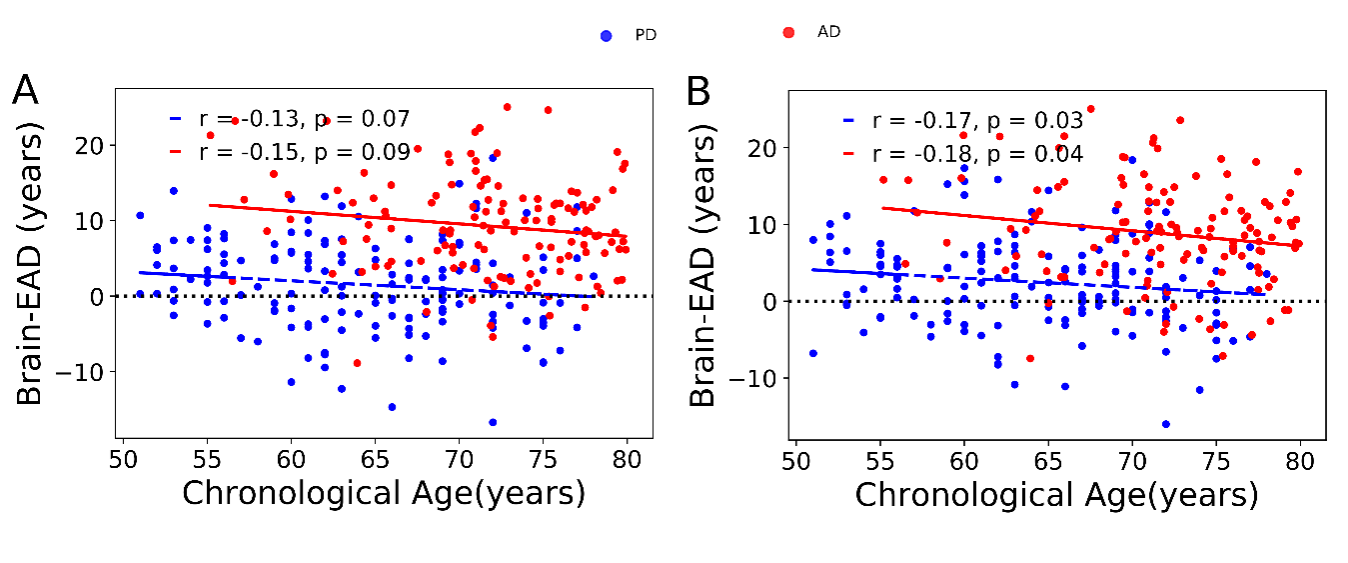
Brain-EAD values versus chronological age among PD patients (blue spot, blue regression line) and AD patients (red spot, red regression line). (A) GM model. (B) WM model. The reference line is illustrated with a dashed black line (y = 0).

The differences and similarities between PD and AD patients have been investigated in a series of studies [27, 36, 37]. Accordingly, we next explored the Brain-EAD in PD versus AD patients. The estimated brain age versus chronological age among PD and AD patients using both the GM and WM models is shown in Figure 5. As reported in Section 3.2, both PD and AD patients showed significantly higher mean Brain-EAD values versus in the training set (PD-GM: +1.50 ± 6.03 years; AD-GM: +9.29 ± 6.43 years; PD-WM: +2.47 ± 5.85 years; and AD-WM: +8.85 ± 6.62 years). According to a paired-samples *t*-test, there was no significant difference between GM Brain-EAD and WM Brain-EAD values (GM mean = 9.29 years; WM mean = 8.85 years; t(128) = 1.08, *p* = 0.28) among AD patients. In contrast, a paired-samples *t*-test indicated that the WM Brain-EAD (mean = 2.47 years) was significantly higher than the GM Brain-EAD (mean = 1.49 years) among PD patients (t(159) = 2.75, *p* = 0.007). As can be seen in Figure 3, there was a significant difference between PD and AD patients in both GM values (mean: 1.50 vs. 9.29 years, F = 39.93, *p* < 0.001, ANCOVA) and WM values (mean: 2.47 vs. 8.85 years, F = 30.51, *p* < 0.001, ANCOVA). Indeed, the AD patients showed significantly higher Brain-EAD values than PD patients in both GM and WM models. The association between Brain-EAD and chronological age for PD and AD groups is illustrated in Figure 6.

### Association between Brain-EAD and clinical parameters

In this section, we present the correlations between Brain-EAD and clinical parameters for both AD and PD patients. Recently, the associations between Brain-EAD and clinical parameters as well as anatomical MRI measurements have been investigated in a series of AD studies [38]. In this study, we explored the association between GM Brain-EAD (because it showed higher values than WM Brain-EAD among AD patients) and related clinical parameters for AD (i.e., MMSE and GDS). In the PD group, we investigated the association between WM Brain-EAD and related clinical parameters for PD (i.e., duration of disease, MoCA, GDS, MDS-UPDRS Total, MDS-UPDRS Part I, MDS-UPDRS Part I Patient Questionnaire, MDS-UPDRS Part II Patient Questionnaire, MDS-UPDRS Part III, S&E, UPSIT, SCOPA-AUT, SBRs of the left/right caudate, and SBRs of the left/right putamen).

Association between Brain-EAD and clinical parameters in the AD group

The results of a partial correlation test between GM Brain-EAD values and respective clinical parameters among AD patients (i.e., MMSE and GDS) with adjustment for age and sex are shown in Table 3. There was no significant correlation between the Brain-EAD and clinical parameters in the AD group.

**Table 3:** Partial correlation (r) of GM Brain-EAD with adjustment for age and sex among AD patients (n = 129)

Variable	r	*p*-value
MMSE	-0.08	0.17
GDS	0.04	0.31

Association between Brain-EAD and clinical parameters in the PD group

**Figure 7.**
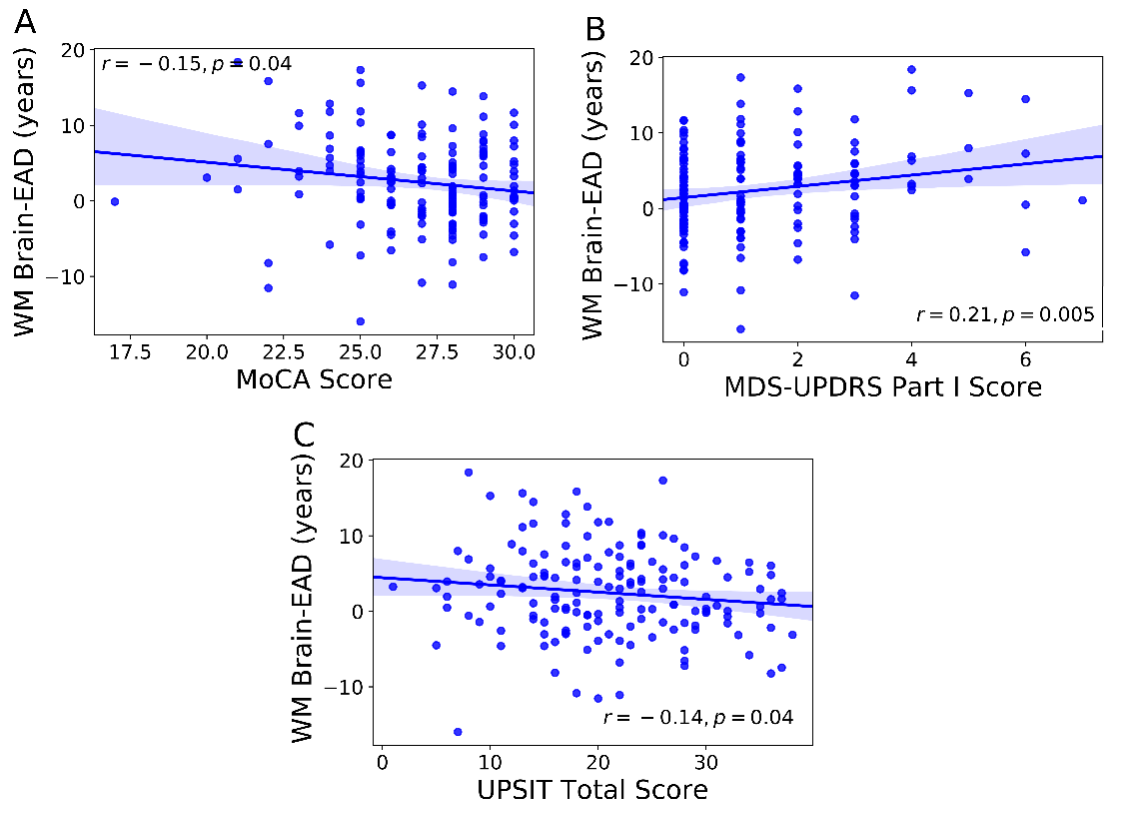
Partial correlations between Brain-EAD results and clinical parameters in the PD group. (A) MoCA, (B) MDS-UPDRS Part I, and (C) UPSIT - Total Score. Variables showing a significant correlation with Brain-EAD are shown.

The associations between the Brain-EAD results and the clinical parameters in the PD group are shown in Table 4. According to the partial correlation test results, the Brain-EAD values showed a relationship with the MoCA, MDS-UPDRS Part I, and UPSIT - Total Score (r = -0.15, r = 0.21, and r = -0.14, respectively; *p < *0.05). There was no significant correlation between the Brain-EAD values and the duration of disease, GDS, MDS-UPDRS Total, MDS-UPDRS Part I, MDS-UPDRS Part I Patient Questionnaire, MDS-UPDRS Part II Patient Questionnaire, MDS-UPDRS Part III, S&E, SCOPA-AUT, SBRs of the left/right caudate, and SBRs of the left/right putamen. The associations between the WM Brain-EAD values and the clinical parameters that were significant among PD patients are shown in Figure 7.

## DISCUSSION

Recent studies have confirmed that the use of neuroimaging-based data followed by multivariate machine learning methods can detect and track brain abnormalities in neuropsychiatric patients. Similarly, several studies have investigated brain age as a reliable biomarker in different brain diseases [15, 17, 22, 28, 31]. For instance, in [25], the researchers modeled a GM-based brain age estimation framework to investigate the brain age values among mild cognitive impairment patients. In another study [31], the researchers investigated the neuroanatomical age estimation for schizophrenia and beyond. They reported an MAE of 4.6 years for HCs and mean brain ages of +1.7, +3.1, +4.0, and +5.5 years for individuals in at-risk mental states for psychosis, borderline personality disorder, major depression, and schizophrenia, respectively.

Similarities and differences between AD and PD have been documented in a series of neurological studies based on behavioral and psychological aspects [3, 39], mechanisms of neurodegeneration [37], and genetics [27]. Accordingly, we conducted this empirical study to explore the Brain-EAD in PD patients and compare it with AD using both GM and WM models. Briefly, our data were derived from 1,128 T1w MRI scans from four different datasets. To the best of our knowledge, this study is the first to estimate brain age through neuroimaging (T1w) data in PD patients. The Brain-EAD value of each individual was determined by subtraction of the brain estimated age from the chronological age. Significant differences in the brain ages of PD and AD patients versus training set were found for both GM and WM models. Regarding the GM model, the mean Brain-EAD values were +1.50, and +9.29 years for PD, and AD individuals, respectively. Our Brain-EAD results are in line with those of a previous brain age study that showed a 10-year increase in Brain-EAD in AD patients on the basis of GM [14]. In this study, we additionally investigated the Brain-EAD on the basis of WM for AD. With respect to the WM model, the mean Brain-EAD values were +2.47, and +8.85 years for PD, and AD individuals, respectively. It is worth noting that a larger positive Brain-EAD value indicates faster brain aging [14]. Thus, higher mean Brain-EAD values among AD patients (for both GM and WM models) indicate significantly advanced brain aging among AD patients than PD patients (Figure 3). Consequently, we can hypothesize that AD patients have a significantly “older-appearing” brain than PD patients, possibly due to greater brain atrophy among AD patients compared with PD patients [40]. In the AD group, although the mean GM Brain-EAD value was higher than the WM Brain-EAD value (mean: +9.29 vs. +8.85 years), there was no significant difference between GM and WM Brain-EAD values among AD patients (t(128) = 1.08, *p* = 0.28, paired-samples *t*-test).

With respect to the PD group, the mean WM Brain-EAD value was higher than the GM Brain-EAD value (+2.47 vs. +1.50 years). Furthermore, our statistical analysis revealed a significant difference between WM Brain-EAD and GM Brain-EAD values among PD patients (t(159) = 2.75, *p* = 0.007, paired-samples *t*-test). Thus, we hypothesized that PD patients would have higher WM Brain-EAD values than GM Brain-EAD values.

We additionally explored the association between the Brain-EAD values and the clinical parameters among AD and PD patients. Among AD patients, there was no significant correlation between Brain-EAD values and the respective clinical parameters (i.e., MMSE and GDS), possibly due to the small range of variables. With respect to PD patients, we observed a significant correlation between Brain-EAD values and the MoCA, MDS-UPDRS Part I, and UPSIT. Given the lack of significant correlations in measures specific to motor symptoms (e.g., MDS-UPDRS Part II and III), the Brain-EAD values appear to be related to non-motor symptoms in PD (e.g., abnormal smelling, dyscognition). Thus, we speculate that Brain-EAD might be a candidate biomarker for non-motor symptoms in PD. However, the correlation values are generally not high, and their significance should thus be carefully interpreted.

**Table 4:** Partial correlation (r) of WM Brain-EAD values with clinical parameters among PD patients with adjustment for age and sex (n = 160).

Variable	r	*p*-value	Variable	r	*p*-value
Duration of disease	-0.06	0.19	S&E	-0.12	0.05
MoCA	-0.15	0.04	UPSIT - Total Score	-0.14	0.04
GDS	-0.13	0.43	SCOPA-AUT	0.02	0.42
MDS-UPDRS Total	0.06	0.23	SBR-Left Caudate	0.01	0.48
MDS-UPDRS Part I	0.21	0.005	SBR-Right Caudate	-0.04	0.27
MDS-UPDRS Part I Patient Questionnaire	0.02	0.42	SBR-Left Putamen	0.02	0.39
MDS-UPDRS Part II Patient Questionnaire	-0.02	0.40	SBR-Right Putamen	-0.12	0.06
MDS-UPDRS Part III	0.06	0.06			

Note: Variables with a significant correlation (i.e., *p* < 0.05) with Brain-EAD are in bold.

In this study, we compared Brain-EAD values between the two most common neurodegenerative diseases (i.e., PD and AD), which show substantial overlap in clinical representation, pathology, and genetics [26, 27]. Nevertheless, some limitations should be considered in this study. First, voxel-wise brain age frameworks require a very large number of T1w MRI scans from HCs in order to build a robust prediction model. To overcome this aspect, the voxel-wise brain age frameworks use the MRI scans from different sites[14, 34, 41]. This point might be considered as a potential weakness, because it was shown that MRI measurements are influenced by scanner characteristics and imaging protocol [42, 43]. However, some of the site effects could be ameliorated using a common pre-processing pipeline for all data (notably, creating a customize DARTEL template). Second, the comparison of different neurodegenerative diseases is complicated because their disease progressions vary, and no common scales can assess them. Finally, the effect of lifestyle was not considered in our brain age frameworks, as it has previously been demonstrated that lifestyle factors such as metabolic syndrome and alcohol abuse in men and healthy liver and kidney functions and an adequate nutrition in women may significantly affect brain aging [24]. We suggest that future studies investigate the associations between behavioral and psychological symptoms of dementia, such as anxiety, depression, apathy, and hallucinations, and brain age estimations for patients with AD and PD.

### Conclusion

In this study, we compared neurological age between AD and PD patients based on robust brain age estimation frameworks with T1w MRI scans. Brain-EAD values were significantly increased in both AD and PD patients compared with the training set, with AD patients showing higher values than PD patients in both GM and WM models. The greater deviation from normality of AD patients suggests that AD patients have a significantly “older-appearing” brain compared with PD patients. We also observed a significant correlation between the Brain-EAD and the MoCA, MDS-UPDRS Part I, and UPSIT.
